# Quantifying implicit biases in refereeing using NBA referees as a testbed

**DOI:** 10.1038/s41598-023-31799-y

**Published:** 2023-03-22

**Authors:** Konstantinos Pelechrinis

**Affiliations:** Department of Informatics and Networked Systems, University of Pittsburgh, Pittsburgh, 15260 USA

**Keywords:** Computational science, Applied mathematics

## Abstract

Implicit biases occur automatically and unintentionally and are particularly present when we have to make *split second* decisions. One such situations appears in refereeing, where referees have to make an instantaneous decision on a potential violation. In this work I revisit and extend some of the existing work on implicit biases in refereeing. In particular, I focus on refereeing in the NBA and examine three different types of implicit bias; (i) home-vs-away bias, (ii) bias towards individual players or teams, and, (iii) racial bias. For this study, I use play-by-play data and data from the Last 2 min reports the league office releases for games that were within 5 points in the last 2 min since the 2015 season. The results indicate that the there is a bias towards the home team—particularly pronounced during the playoffs—but it has been reduced since the COVID-19 pandemic. Furthermore, there is robust statistical evidence that specific players benefit from referee decisions more than expected from pure chance. However, I find no evidence of negative bias towards individual players, or towards specific teams. Finally, my analysis on racial bias indicates the absence of any bias.

## Introduction

Being a referee in sports is without question a very tough job. There are decisions that need to be made in literally a split second, and are required to make these decisions with high accuracy. On top of this, they have to endure almost constant complaints from the two teams being refereed. When having to make decisions this quickly, the human brain has to rely on various heuristics and this is where implicit bias can get into the way of judgement^1^. Referees—like all humans—are not immune to these type of biases and prior work has reported on a variety of similar instances. For example, baseball umpires exhibit the gambler’s fallacy in the call of pitches, showing a negative auto-correlation in their calls of consecutive ambiguous pitches^2^. Umpires also exhibit higher error rate when there were 3 balls or 2 strikes (excluding full counts), favoring the call that would not end the at bat^3^. This is a result of another cognitive shortcut, namely, impact aversion, which is essentially a bias towards doing nothing. Price and Wolfers^4^ using foul data from the NBA for the seasons between 1992 and 2004 found that on average players get called for more fouls when officiated by an opposite-race crew as compared to when being officiated by a same-race crew. This study steered a lot of discussion in the league office and in 2010 Pope, Price and Wolfers^5^ revisited the question and analyzed data over two 3-year periods, one before the publication of the original study (2003–2006) and one after (2007–2010). They found that during the first period there was still a significant racial bias in calling fouls, while this bias was no longer present in the second period. This is a valuable finding, since it provides evidence that the knowledge of implicit biases can help in reducing or even eliminating them. More recently Mocan and Osborne-Christenson^6^, using data from the NBA’s last 2 min reports (to be described later) did not find any biases with regards to incorrectly called fouls, but there were significant in-group biases with regards to non-called fouls. Referee bias is also considered to be one of—if not the—major reasons for home field advantage^3^. Earlier studies on this had utilized only a handful of games played without fans because of sanctions imposed on teams, and the results were mixed^7,8^. Many recent studies utilized the natural experiment setting provided by the COVID-19 pandemic to examine the impact of empty arenas and stadiums on the home court advantage and refereeing bias^9–15^, with the majority of them pointing to a negative impact of reduced or no crowds on home field advantage and officials bias towards the home team^10–12,16^. Referees might not only show a bias towards the home team but also towards specific individual—*star*—players or specific teams, regardless of where the game is played (e.g., teams that are on the top of the standings). While the volume of research for this type of biases is undoubtedly smaller, there are studies that have examined them. For example, Barrett^17^ found that players with higher salary (a proxy for the star-quality of a player) receive more fouls drawn calls per 48 min, while Caudil et al.^18^ found that NBA All Stars are awarded with an additional 0.32 free attempts per minute during the fourth quarter of NBA Playoff games. In a different sport, Findlay and Ste-Marie^19^ found that figure skaters known to the judges received higher marks as compared to unknown athletes. Erikstad and Johansen^20^ analyzed penalty data from the Norwegian league and found that successful teams were more likely to receive an incorrect penalty compared with their opponents, and less likely to be denied a penalty they should have been awarded. Nevertheless, there are contradicting studies with null results, i.e., no signs of player or team bias. For example Morgulev et al.^21^ did not find any bias with regards to star players and teams in the NBA, while Bose et al.^22^ examined the presence of a “status” team bias in the German soccer top-league but they were not able to identify any. A tangential line of research has also looked at the underlying mechanisms that lead to similar potential biases. I elaborate on the related literature in the last section, where I also connect this work, its findings and methods to the existing literature.

The objective in this work is to examine possible implicit biases in NBA refereeing at three different levels: (i) home vs away teams, (ii) individual (super star) players or teams, and, (iii) players/referee race. I use play-by-play data as well as data from the Last Two Minute (L2M) reports since the 2015 season for this study. The L2M reports include a detailed break down of the events that took place during the last 2 min of *close* games, defined as games within 5 points. There is an entry for each call on whether the call was correct or not. There is also information about which player/team benefited/disadvantaged from this call. Furthermore, there is the same information on missed call. For studying the first two types of implicit bias I use the L2M reports and estimate the net whistle gain for a team or player based on the situations where they benefited or were disadvantages. I further empirically estimate its statistical significance through Monte Carlo simulations. For the racial bias I make use of play-by-play data since the L2M reports do not have information with regards to which referee made the call. As I elaborate more at the Supplementary Material, even if I was able to overcome the inconsistencies between the two data sources (mainly with regards to the game clock) and match the L2M data with play-by-play and obtain information about which referee made a call, only 3% of the foul calls (a total of 210) is incorrect. On the contrary while there are more incorrect non-calls when it comes to fouls (a total of 1399 for approximately 12% of all fouls) it is impossible to know which referee was responsible for the call (e.g., the closest referee). Thus, I will use play-by-play data similar to previous studies^4,5^. However, unlike prior studies, I am not relying on foul calls. In order to properly analyze these calls one needs to consider which player benefited from the call as well, a piece of information missing from prior analysis and one that it is not available for all foul calls from play-by-play data. Furthermore, given that only a very small of the fouls called is incorrect, then this could bias the calculations as most of the calls are correct and therefore, needed to be made. Hence, I rely on analyzing the technical fouls called from referees, which are also more subjective compared to foul calls.

The main findings can be summarized in the following:

- During the whole period that the data cover there is overall a home-team bias, which is even more pronounced during the playoffs. However, this bias has almost been eliminated since the 2020 season.
- There are specific players that exhibit a statistically significant positive net whistle gain. However, the same is not true for the opposite direction, i.e., players that have a statistically significant negative net whistle gain.
- I do not find evidence of bias in any direction towards individual teams.
- There is not any racial bias observed when analyzing (personal) technical fouls called.

The rest of this paper is organized as follows: In the following section I present in detail the data used and the analysis methods. Next I present and discuss the results, while in the last section I discuss existing relevant literature and its connection with this study, while I also conclude the work, discussing its limitations and future steps.

## Methods

For this study I used the L2M reports data covering the seasons between 2015 (the first season the NBA started releasing the reports) until this past season 2022. The data were collected and are made publicly available at the following github repo: https://github.com/atlhawksfanatic/L2M. Each entry in the L2M includes several elements but the ones that I make use of in the analysis are: committing player, disadvantaged player, committing side, disadvantaged side, decision. The decision takes 4 possible values: correct call (CC), incorrect call (IC), incorrect non-call (INC) and correct non-call (CNC). While CC, IC and INC decisions are well-defined, CNC decisions are not. In theory, every second in the game with no violation is a CNC. Hence, the instances included in the reports are subjective and the criteria can change from year-to-year. In fact, during the 2015 season there were 6.4 CNC entries per game, while during the 2022 season there were almost 14 CNC entries per game. This means that any analysis should not rely on CNC data points since they are not consistent across seasons.

I also collect the play-by-play data through the NBA API. These data provide information for the events that took place during each game, including the technical fouls called. I only consider personal technical fouls, that is, I filter out calls like defensive 3 s, delay of game etc., that are labeled as technical fouls as well. For every technical foul the play-by-play data also provide information for the referee calling it and of course the player receiving it. I further collected the demographics of the referees manually, i.e., going over their profiles on the league’s webpage^23^ while for players I used an online database with racial information about the players^24^. For those players not in the database I followed the same procedure as with the referees, by visiting their profile page on the league’s webpage.

### Home court

To examine possible home-court biases in refereeing I start by calculating the home team net whistle gain for all the games in the L2M dataset. The net whistle gain for the home team consists of two parts, namely, the *whistle benefit* and the *whistle detriment*. The whistle benefit $\beta$ is just the number of INC decisions when the committing side is the home team plus the number of IC decisions when the committing side is the visiting team. Similarly, the whistle detriment $\delta$ for the home team is the number of INC decisions when the committing side is the visiting team plus the number of IC decisions when the committing side is the home team. Then the net whistle benefit $w_g$ for the home team is simply $w_g= \beta -\delta$. This is essentially the total number of times that the home team benefited from the referee decision. If $w_g> 0$ the home team got overall the “better whistle”, while if $w_g< 0$ the visiting team got the better whistle.

**Figure 1:**
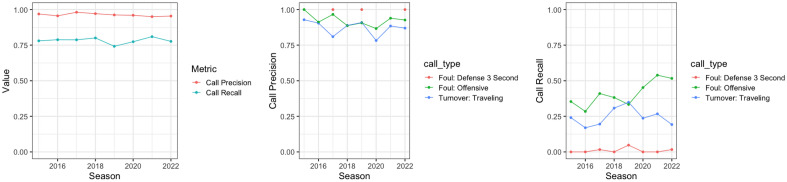
Precision and recall overall and of different violation types over the last 8 seasons.

However, the question is whether $w_g$ is statistically different than zero or we could have expected this by the stroke of luck. In order to answer this question I rely on Monte Carlo simulations. In particular, I simulate the decisions on all actual violations and calls in the dataset based on the precision and recall rates of violations. I define as the violation calls precision as the ratio: $\dfrac{CC}{CC+IC}$, while the recall of a violation is: $\dfrac{CC}{CC+INC}$. Given that not all violation types have the same precision or recall I calculate these metrics separately for each violation type. Figure 1 (left), shows the overall precision and recall for all violations over the seasons covered in the data. It is evident that when a call is made, this is a true violation with very high probability ($> 95\%$). Nevertheless, there is only about 80% recall rate, that is, about 20% of the true violations are missed. The middle and right parts of Figure 1 further show the differences in precision and recall rates for different violations (Table 1 provides the precision and recall rates for all types of violations, while in the Supplementary Material I provide the yearly precision and recall for violations with at least 200 data points over the period covered from the data). There are some striking observations. For instance, almost none of the defensive 3 s violations is being called in the last 2 min of close contests (low recall), while about 15% of traveling calls are incorrect (precision $\approx 85\%$). Through the discrete event simulations I can estimate the empirical distribution for $w_g$, $\hat{f}_{w_g}$, and this will allow us to estimate the empirical p-value for $w_g$. In what follows I provide some details on the core of the simulation engine.

**Figure 2:**
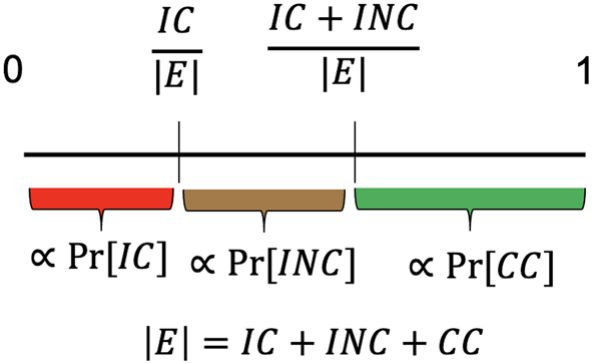
Decision boundaries for the simulation of the calls.

An event for this study is a made call (correct or not) or an actual violation (called or not). This means that the total number of events I simulate is essentially $CC+INC+IC$. For every event a decision has to be made on whether a correct call was made (CC), or an incorrect call was made (IC) or a violation was erroneously not called (INC). The probability of each one of these events is proportional to the corresponding base rate. Therefore, for every call I draw a uniformly distributed random number *r* between 0 and 1 and I have the following decision boundaries (see Fig. 2):

- If $r \in [0,\dfrac{IC}{IC+INC+CC})$, there is an incorrect call.
- If $r \in [\dfrac{IC}{IC+INC+CC},\dfrac{IC+INC}{IC+INC+CC})$, there is an incorrect non call.
- If $r \in [\dfrac{IC+INC}{IC+INC+CC}, 1]$, there is a correct call.

Given that I should treat each type of violation/call differently, the decision boundaries are different for every type of violation. This allows us to control in the simulations for the “difficulty” of the violations a team is involved in.

### Player and team-specific

For examining the presence of player-specific implicit biases by the referees, I use the same method as above. However, given that I essentially perform multiple statistical tests—one for each player—I expect some of them to deem statistically significant results even by chance. Therefore, I perform a meta-test to calculate the probability that all of the data points that came out as statistically significant are false positives^25,26^. In particular, under the—realistic in this case—assumption that the tests are not correlated I can use the Binomial distribution for a meta-test. With *M* tests each of which has a probability of $\alpha$ leading to a false positive result, I can estimate the probability of observing at least *r* positive tests due to chance as: $$\sum _{p=r}^{M} \left( {\begin{array}{c}M\\ p\end{array}}\right) \alpha ^p (1-\alpha )^{M-p}$$. If this probability is small, then one can confidently concludes that the non-zero effect sizes observed are not all false positives. I follow exactly the same approach for examining team-specific biases.

### Racial bias

The last type of implicit bias that I examine is that of race, which, is an instance of the affect bias/heuristic^27^. As aforementioned I will rely on the technical fouls called and in particular I will compare (a) the call rate of technical fouls to players of the same race as the referee $\tau _{same}$ making the call with, (b) the call rate to players of different race $\tau _{diff}$. This requires the computation of not only the number of technical fouls within and across races, but also of the total minutes that a referee was on the court with players of the same and different race^28^. In order to estimate the statistical significant of the difference $\Delta \tau =\tau _{diff}-\tau _{same}$, I rely again on Monte Carlo simulation. In particular, for every referee I estimate their overall call rate per game for technical fouls. I then iterate over every game they refereed and perform a two-step simulation. First, based on the referee’s call rate I simulate the binary decision on whether the referee called a simulated technical foul in the game or not. Second, if a technical foul is simulated, the recipient is randomly chosen among the players that took the court in the game. The probability of a player receiving the simulated technical foul is proportional to their playing time in the game. By repeating this process several times I can obtain the empirical distribution $\hat{f}_{\Delta \tau }$ under the null hypothesis that there is no racial bias (controlling for the racial composition of players and referees in the various games).

**Table 1:** Precision and recall of different types of violation.

Violation	Precision	Recall	N	Violation	Precision	Recall	N
Turnover: traveling	0.85	0.24	692	Turnover: stepped out of bounds	0.92	0.77	169
Foul: personal	0.97	0.90	7498	Turnover: kicked ball violation	0.55	0.55	16
Turnover: 8 s violation	0.81	0.73	35	Foul: away from play	0.84	0.45	117
Turnover: out of bounds	0.69	0.46	47	Foul: personal take	0.99	0.99	702
Foul: shooting	0.93	0.79	4433	Foul: punching	0.55	0.60	15
Stoppage: out-of-bounds	0.91	0.97	299	Stoppage: other	0.44	0.57	12
Foul: loose ball	0.95	0.54	1162	Foul: delay technical	0.71	0.79	25
Instant replay: support ruling	0.99	1.00	858	Turnover: 10 s violation	0.50	0.42	17
Foul: double personal	0.50	0.38	22	Turnover: discontinue dribble	0.40	0.22	24
Foul: offensive	0.91	0.40	1120	Violation: other	0.43	0.55	19
Turnover: 24 s violation	0.98	0.96	347	Instant replay: support	0.55	0.67	14
Instant replay: overturn ruling	0.98	0.99	302	Foul: inbound	0.50	0.42	17
Foul: technical	0.95	0.92	114	Turnover: lane violation	0.67	0.71	19
Foul: double technical	0.76	0.84	24	Turnover: 5 s violation	0.86	0.53	78
Ejection: second technical	0.62	0.73	16	Turnover: inbound turnover	0.55	0.38	21
Turnover: offensive goaltending	0.87	0.73	50	Turnover: punched ball	0.44	0.50	13
Violation: kicked ball	0.92	0.85	127	Instant replay: overturn	0.50	0.62	13
Turnover: 3 s violation	0.65	0.10	137	Turnover: illegal assist	0.44	0.50	13
Turnover: backcourt turnover	0.84	0.81	60	Turnover: lost ball out of bounds	0.94	0.96	202
Violation: jump ball	0.80	0.83	29	Violation: free throw	0.50	0.62	13
Violation: lane	0.87	0.36	97	Turnover: out of bounds − bad pass	0.97	0.99	208
Turnover: 5 s inbound	0.72	0.36	66	Foul: shooting foul	0.44	0.57	12
Turnover: jump ball violation	0.62	0.62	18	Foul: hanging technical	0.44	0.50	13
Foul: flagrant type 1	0.79	0.86	27	Foul: offensive charge	0.64	0.75	17
Violation: defensive goaltending	0.94	0.81	100	Foul: personal block	0.44	0.50	13
Foul: defense 3 s	0.58	0.03	273	Foul: shooting block	0.44	0.57	12
Turnover: lost ball possession	0.55	0.60	15	Turnover: bad pass	0.62	0.73	16
Turnover: double dribble	0.74	0.44	45	Turnover: foul	0.50	0.62	13
Violation: delay of game	0.92	0.80	79	Turnover: lost ball	0.64	0.75	17
Turnover: palming	0.54	0.37	25	Free throw technical	0.44	0.50	13
Turnover: illegal screen	0.55	0.50	17	Stoppage: TimeOut	0.54	0.64	17
Foul: clear path	0.85	0.90	36	Stoppage: clock	0.55	0.60	15
Violation: double lane	0.55	0.33	23	Foul: defensive 3 s	0.44	0.50	13

## Results

### There is referee home court bias, but it has been small since the COVID-19 pandemic

I start by looking at the L2M data and estimating the net whistle gain for the home team during the whole period covered in the data. Table 2 presents the results, where we can see that there is overall a statistically significant home court referee bias, with the home team having benefited in approximately 146 situations more than expected. This corresponds to an 1.2 percentage units difference between the home and visiting team. Furthermore, as we can see the home court bias is much higher during the playoffs. Given that home court referee bias is part of the home court advantage (HCA), which has been linked to the home team fans, I wanted to examine separately the seasons during/after the COVID-19 pandemic. The NBA finished the 2020 season in a bubble with no fans, and started the 2021 season in empty arenas. In fact, most of the teams didn’t start having fans at limited capacity until the middle of that season and only reached arenas with fans closer to capacity during the playoffs. As we can see from Table 2 the home court referee bias appears to be very small, and almost have disappeared during these seasons! This is in fact in agreement with the point-equivalent of the overall home court advantage as estimated from team regression ratings. In particular, based on the Sagarin ratings^29^ the home court advantage between 2015 and 2019 was 2.74 points, while between 2020 and 2022 it dropped to 1.75 points.

So overall, we see the presence of a home team referee bias. However, in the second time period I analyzed this bias has a lower magnitude. It remains to be seen whether this has been an artifact of empty arenas during the COVID-19 pandemic. For example, one other possible mechanism that can have (at least partially) led to this diminished home court advantage in the second time period, is the introduction of coaches challenge in the 2020 season, where a coach can contest one call per game. This triggers an automatic review and the call can change. When NFL introduced a similar system the win percentage of the home teams dropped from 58.5 to 56%^3^. However, in the NFL coaches can have up to 3 challenges, while in the NBA it is strictly 1. Another difference is the fact that in the NBA coaches can only challenge made calls (e.g., a foul called), and not missed calls (e.g., a foul that was not called). As we saw in Fig. 1, the majority of the referee mistakes originate from non-calls rather than calls. Therefore, the impact is expected to be overall smaller, but nevertheless there are several anecdotes supporting its possible impact on the home court advantage. For example, during the very first week of this new rule, Portland won in Dallas to a large degree due to a coaches challenge that overturned a foul called 5 s before the end of the game against Portland. This call overturn resulted in a 34 percentage unit swing in the win probability in favor of Portland according to ESPNs win probability model^30^.

The home court advantage appears to be particularly pronounced in the playoffs (Table 2). While in the playoffs the “better” team (according to the league standings in the regular season), plays in general more games at home, the analysis includes all games, i.e., even games where the home team was the “worse” of the two. This means that the major reason that drives the “better whistle” is not the quality of the team but the fact that one team plays at its home court. Of course, there can be many additional reasons that lead to the pronounced playoff home field advantage, but identifying these causes is beyond the scope of this work. For example, one plausible additional mechanism is that referees are averse to making an erroneous call or missing a call that leads closer to a team losing the series, and, thus getting disqualified from the playoffs. This situations appear the majority of the cases when the home team is the lower seed, and hence, they might be getting even more beneficial whistle than what they would get in a regular season game.

**Table 2:** The home court bias has reduced since the 2020 season, which is the season of the COVID-19 pandemic.

Seasons	Season type	p-val	$w_g- \textbf{E}[\hat{f}_{w_g}]$ (%)
2015–2022	Regular	0.03	107.7 (1.2%)
2015–2022	Playoffs	< 0.01	47.86 (7%)
2015–2022	Both	< 0.01	145.55 (1.6%)
2015–2019	Regular	0.02	97.45 (1.5%)
2015–2019	Playoffs	< 0.01	41.12 (9.4%)
2015–2019	Both	< 0.01	142.15 (2.2%)
2020–2022	Regular	0.45	2.97 (0.02%)
2020–2022	Playoffs	0.26	6.26 (2%)
2020–2022	Both	0.49	8.94 (0.02%)

### There is player-specific bias, but only positive. There is no team-specific bias

Next I examine the net whistle gain for individual players over the seasons covered from the L2M data. I repeat the same process as for the home court referee bias, but now focusing on individual players. I only use in the analysis players that have been involved in at least 100 calls/missed calls over the whole 8-year period (this corresponds to the top 10th percentile). This provides us with a total of 106 players. Also when estimating the base call/miss rate for a violation type I filter out the data of the specific player I simulate. Table 3 shows the results for all players where we can see that there are 12 players that exhibit a statistically significant positive net whistle gain (at the 5% significance level). Using the binomial metatest aforementioned, there is an approximately 7-in-1000 chance that all of these 12 instances are false positives. Therefore, we can say with quiet some confidence that there are specific players that get a “better whistle” than expected. We can also see that most of these players are all-stars, all-NBA and/or all-defensive NBA players (e.g., Dwyane Wade, Chris Paul, Carmelo Anthony, Karl-Anthony Towns, Jayson Tatum, Andre Drummond, Hassan Whiteside, Patrick Beverley). I also looked at the opposite direction, i.e., whether there are players that consistently get a “worse whistle” than expected. There is a total of 7 players that exhibit a statistically significant negative net whistle gain. However, the probability of all of these 7 instances being false positives is non-negligible and equal to 28%.

Turning to the team-specific analysis, Table 4 depicts the results, where as we can see there are a few (3) teams that have a positive net whistle gain and a few (3) teams that have a negative net whistle gain. However, the probability that all of these cases are false positives, is non-negligible as well (19%). Overall, we can say that the data support the presence of a player-specific referee bias. However, it is only in one direction, that is, specific players benefiting more than expected. Furthermore, the composition of the group of players that exhibit the positive net whistle gain points to a bias towards “star” players. Nevertheless, given the fact that there are other star players that do not experience the same net benefit it is hard to argue that this is explicitly the reason behind any implicit bias observed. Finally, there were no strong evidence of team-specific bias.

### There is no evidence of racial bias observed among NBA referees

**Figure 3:**
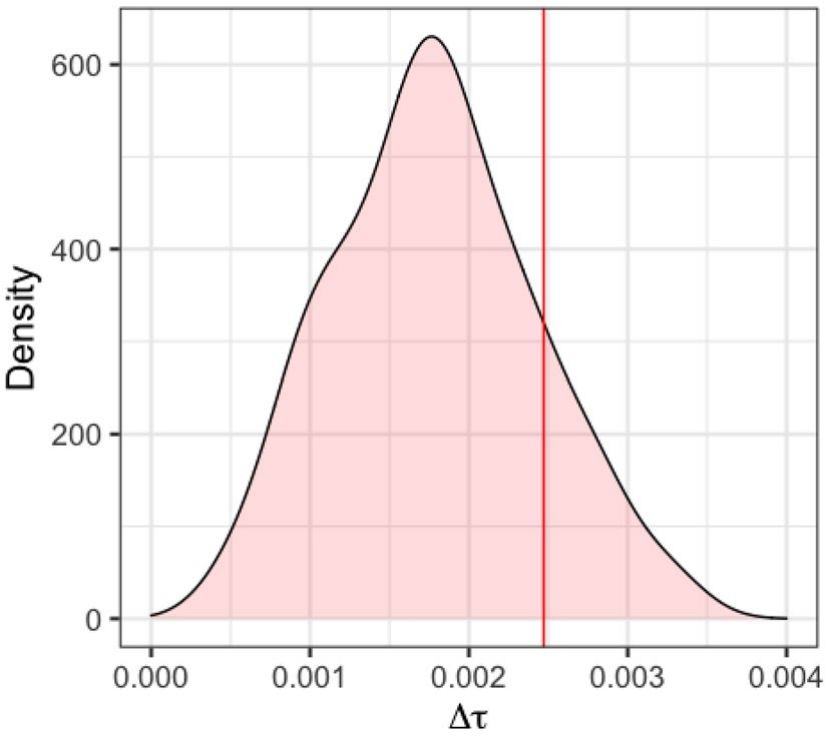
The difference in the personal technical fouls call rate between same and different referee-player race is not statistically different than the one expected by random chance.

Lastly I examine the presence of racial bias in refereeing decisions. Given that 92% of both the referees and players in the data are white or African American, I focus on these two racial groups in the analysis. I also filter out the games for which there is no available information for all the referees. These situations correspond to about 3.6% of the games. In the dataset, there are 5419 (personal) technical fouls called. There were 0.0204 technical fouls per 48 min called from referees to opposite race players, while, referees called 0.0182 techs per 48 min to players of the same race. So overall, referees called 0.0022 more technical fouls per 48 min to players of the opposite race as compared to players the same race as them. This difference by itself, even if statistically significant, is hard to be qualified as racial bias, since it corresponds to 1 more technical foul per 450 games approximately. Furthermore, I estimated the distribution of tech call rate difference $\Delta \tau$ through simulating the technical fouls as described earlier. Figure 3 presents the distribution, while the vertical line corresponds to the actual tech call rate difference obtained from the real data. In the simulations, I obtained a value of $\Delta \tau >0.0022$ in 33% of the cases, indicating that even the small difference observed is not statistically significant. This is in agreement with the latest study by Pope, Price, and Wolfers^5^, providing additional evidence, that is, through examination of different violation calls, for the absence of implicit racial bias by the NBA referees.

**Table 3:** Net whistle gain for individual players.

	Player	$w_g- \textbf{E}[\hat{f}_{w_g}]$ (%)	pval		Player	$w_g- \textbf{E}[\hat{f}_{w_g}]$ (%)	pval
1	Harrison Barnes	2.88 (1.93%)	0.32	54	Dwight Howard	7.05 (6.91%)	0.09
2	Isaiah Thomas	− 1.295 (− 1.04%)	0.65	55	Nikola Vucevic	− 4.525 (− 2.85%)	0.83
3	Stephen Curry	4.975 (2.29%)	0.23	56	Kyle Lowry	8.48 (3.64%)	0.08
4	Danilo Gallinari	2.75 (2.43%)	0.33	57	Tim Hardaway Jr.	3.225 (3.1%)	0.23
5	James Harden	10.065 (2.48%)	0.14	58	**Chris Paul**	10.905 (3.97%)	0.04
6	LeBron James	− 9.35 (− 3.38%)	0.92	59	Blake Griffin	1.35 (0.83%)	0.42
7	Brook Lopez	2.145 (1.69%)	0.34	60	Jrue Holiday	− 6.145 (− 3.09%)	0.91
8	Andrew Wiggins	− 8.44 (− 4.72%)	0.96	61	Draymond Green	5.585 (3.12%)	0.14
9	JJ Redick	− 0.99 (− 0.82%)	0.58	62	Al Horford	6.06 (3.94%)	0.15
10	DeAndre Jordan	1.46 (1.11%)	0.39	63	Kevin Durant	− 3.225 (− 1.84%)	0.77
11	Paul Millsap	1.56 (1.11%)	0.40	64	Paul George	2.25 (1%)	0.40
12	Jeff Teague	2.035 (1.65%)	0.36	65	Jonas Valanciunas	0.835 (0.75%)	0.47
13	**Dennis Schroder**	10.965 (5.96%)	0.02	66	Aaron Gordon	− 2.035 (− 1.88%)	0.70
14	LaMarcus Aldridge	− 0.43 (− 0.27%)	0.56	67	**Steven Adams**	10.65 (6.16%)	0.04
15	Nicolas Batum	− 4.99 (− 4.94%)	0.91	68	**Carmelo Anthony**	6.825 (6.5%)	0.05
16	Wesley Matthews	− 2.125 (− 1.7%)	0.74	69	Evan Fournier	− 4.145 (− 2.84%)	0.84
17	Damian Lillard	− 4.165 (− 1.46%)	0.79	70	Ricky Rubio	− 0.96 (− 0.62%)	0.64
18	**Dwyane Wade**	9.72 (6.89%)	0.04	71	Julius Randle	− 5.48 (− 2.19%)	0.82
19	**Hassan Whiteside**	10.44 (9.67%)	0.01	72	Kristaps Porzingis	− 1.45 (− 1.25%)	0.66
20	Anthony Davis	2.74 (1.36%)	0.33	73	**Karl-Anthony Towns**	13.295 (6.04%)	0.05
21	Russell Westbrook	3.41 (0.97%)	0.33	74	**Cody Zeller**	9.805 (9.43%)	0.02
22	Kyrie Irving	2.67 (1.47%)	0.36	75	Goran Dragic	1.88 (1.17%)	0.39
23	Marcus Morris	6.57 (4.73%)	0.10	76	**Mason Plumlee**	11 (10.28%)	0.00
24	PJ Tucker	2.905 (1.73%)	0.29	77	Serge Ibaka	5.855 (5.32%)	0.12
25	Eric Bledsoe	3.995 (3.1%)	0.23	78	**Patrick Beverley**	9.66 (8.7%)	0.01
26	Mike Conley	− 5.405 (− 3.02%)	0.89	79	Nikola Jokic	− 12.485 (− 4.59%)	0.95
27	Marc Gasol	5.8 (3.67%)	0.17	80	Gary Harris	2.715 (2.45%)	0.30
28	DeMarcus Cousins	− 1.59 (− 1.02%)	0.66	81	Jusuf Nurkic	4.775 (4.01%)	0.14
29	Tobias Harris	4.035 (2.48%)	0.21	82	Devin Booker	0.79 (0.33%)	0.47
30	Bradley Beal	1.65 (0.72%)	0.44	83	Bojan Bogdanovic	− 1.785 (− 1.65%)	0.69
31	John Wall	− 2.27 (− 1.47%)	0.67	84	Myles Turner	1.06 (0.88%)	0.47
32	George Hill	− 0.13 (− 0.1%)	0.55	85	D’Angelo Russell	0.62 (0.55%)	0.50
33	Kent Bazemore	0.58 (0.44%)	0.50	86	Spencer Dinwiddie	− 1.67 (− 1.11%)	0.68
34	Marcus Smart	7.615 (3.95%)	0.09	87	Josh Richardson	− 2.025 (− 1.35%)	0.70
35	Khris Middleton	− 5.84 (− 2.86%)	0.84	88	Joel Embiid	− 2.38 (− 1.03%)	0.67
36	Jerami Grant	− 1.51 (− 1.14%)	0.68	89	Brandon Ingram	− 2.235 (− 1.73%)	0.75
37	Robert Covington	0.38 (0.26%)	0.56	90	Jamal Murray	− 8.385 (− 6.35%)	0.97
38	Kawhi Leonard	0.70 (0.45%)	0.47	91	Kelly Oubre	4.73 (4.68%)	0.17
39	Elfrid Payton	− 7.94 (− 7.86%)	0.97	92	Malcolm Brogdon	0.875 (0.84%)	0.48
40	Gordon Hayward	− 7.495 (− 5.77%)	0.95	93	Buddy Hield	− 1.04 (− 0.61%)	0.63
41	Rudy Gobert	1.215 (0.49%)	0.47	94	Caris LeVert	− 7.33 (− 6.85%)	0.98
42	Will Barton	− 7.605 (− 5.21%)	0.95	95	Jaylen Brown	− 0.01 (− 0.01%)	0.56
43	Zach LaVine	0.65 (0.29%)	0.55	96	Fred VanVleet	2.645 (2.62%)	0.31
44	Giannis Antetokounmpo	− 3.175 (− 1.04%)	0.68	97	Ben Simmons	− 0.585 (− 0.48%)	0.63
45	Reggie Jackson	1.26 (0.64%)	0.41	98	De’Aaron Fox	2.895 (1.84%)	0.36
46	Jae Crowder	0.43 (0.34%)	0.53	99	**Jayson Tatum**	8.365 (4.78%)	0.04
47	Kentavious Caldwell-Pope	2.63 (2.05%)	0.30	100	Donovan Mitchell	− 4.815 (− 2.75%)	0.86
48	**Andre Drummond**	12.745 (7.92%)	0.01	101	Bam Adebayo	7.42 (5.38%)	0.08
49	Kemba Walker	− 2.425 (− 0.99%)	0.68	102	Domantas Sabonis	6.075 (5.15%)	0.14
50	CJ McCollum	− 1.63 (− 0.95%)	0.62	103	Pascal Siakam	− 5.895 (− 3.88%)	0.89
51	DeMar DeRozan	− 3.35 (− 1.08%)	0.69	104	Luka Doncic	− 6.18 (− 5.72%)	0.94
52	Victor Oladipo	− 0.29 (− 0.21%)	0.56	105	Trae Young	2.16 (1.61%)	0.42
53	Jimmy Butler	3.545 (1.33%)	0.33	106	Ja Morant	2.15 (1.81%)	0.37

Significant values are in bold.

**Table 4:** Net whistle gain for different teams.

	Team	$w_g- \textbf{E}[\hat{f}_{w_g}]$ (%)	pval		Team	$w_g- \textbf{E}[\hat{f}_{w_g}]$ (%)	pval
1	GSW	3.73 (0%)	0.41	16	POR	17.75 (0.02%)	0.14
2	BOS	17.79 (0.01%)	0.14	17	MIA	25.24 (0.02%)	0.04
3	NOP	5.26 (0%)	0.31	18	PHI	− 9.79 (− 0.01%)	0.75
4	DEN	− 26.46 (− 0.02%)	0.97	19	OKC	22.06 (0.02%)	0.07
5	HOU	6 (0.01%)	0.32	20	PHX	− 0.37 (0%)	0.48
6	CLE	− 17.36 (− 0.02%)	0.92	21	SAC	7.99 (0.01%)	0.28
7	BKN	− 19.3 (− 0.02%)	0.92	22	ORL	− 9.34 (− 0.01%)	0.79
8	MIN	13.89 (0.01%)	0.14	23	MIL	− 29.02 (− 0.03%)	1.00
9	LAC	10.18 (0.01%)	0.30	24	IND	22.16 (0.02%)	0.06
10	ATL	− 6.37 (− 0.01%)	0.67	25	NYK	− 19.25 (− 0.02%)	0.92
11	CHI	− 24.7 (− 0.02%)	0.94	26	DET	24.56 (0.02%)	0.04
12	WAS	13.27 (0.01%)	0.15	27	UTA	− 30.14 (− 0.03%)	1.00
13	CHA	− 0.93 (0%)	0.48	28	SAS	16.26 (0.02%)	0.14
14	LAL	− 7.17 (− 0.01%)	0.76	29	DAL	6.02 (0.01%)	0.29
15	MEM	31.25 (0.03%)	0.00	30	TOR	16.27 (0.01%)	0.15

## Related work and discussion

As I mentioned at the introduction, there are different lines of literature that have explored the presence of various types of referee biases, notably home court, “star” player and racial biases^4,5,9–15,19–22^ in a variety of sports. Almost all of these studies use as the variable of interest the volume of calls (or some function of it) for or against teams/players. This makes the implicit assumption that all the violation calls are correct, while no violation call was missed, which is not true as we saw from the analysis of L2M. For example, in the studies on racial bias by Price, Pope and Wolfers^4,5^ the fouls called were analyzed, but the correctness of the call or not was not available and hence, not included in the analysis. Similarly, actual foul calls that were missed was not possible to be included in the study. One way to overcome this problem is to rely on calls that are highly subjective, such as technical fouls (e.g., for excessive complaining, arguing with the referees or other players etc.). Another way to solve the problem identified above is to use knowledge of the correctness or not of a call (or missed call). While some studies have recruited experts to help them annotate correct/incorrect calls and non-calls^20,22,31^, they are inevitably small-scale due to the manual labor associated with annotating a game. However, the L2M data allow for a larger scale analysis. The L2M data I used for the analysis provide this opportunity and the work by Mocan and Osborne-Christenson^6^ is the only one that has made use of this information. Nevertheless, they focus only on fouls, which is only part of the violations committed in a game. By using technical fouls and data from the L2M reports, this work expands on prior literature on the identification and quantification of referee biases by providing additional evidence for the presence or lack of the different types of biases examined.

To summarize, I analyzed L2M and play-by-play data from the NBA to analyze a number of different implicit biases that the referees might exhibit. I started by looking at the home court bias and I found that while over the past 7 years there is a robust home court advantage, this has been in the decline over the last few years. However, given that this period overlaps with the COVID-19 pandemic, and the absence of fans from the arenas, it remains to be seen whether this observation is a trend or an anomaly, since various studies have shown that the absence of fans is related with the reduction in home court/field advantage^10–12,16^. I then examined the possibility of player and team-specific bias. My analysis indicates that there is evidence for the presence of a bias that is driven by players (not teams) and only in the positive direction (i.e., specific players benefiting more than expected from the calls or non calls). Finally, I examined the presence of racial bias in the referee decisions using the personal technical fouls called as a proxy, and I did not find any evidence of racial bias. A key part of the analysis is the simulation of the calls/violations recorded in the L2M data. This requires the estimation of the call base rates for each violation type. These base rates might be noisy when only a very little amount of data are available for a given type of violation. While this in general can be problematic, in this case I do not expect this to affect the results since these violation types will also not appear in the simulations frequently. Nevertheless, an alternative is to use the Bayesian average for the decision boundaries.

One tangential line of research deals with the underlying mechanisms and mental models related to the decision making of referees. A variety of processes have been proposed and discussed in the literature. For instance, compensation strategies^32^ lead to referees making decisions on ambiguous situations by considering previous calls. For example, if a soccer referee has already awarded a penalty kick to a team, s/he is less possible to do again in an ambiguous call for the same team but more probable to call the penalty for the opposing team. Game management strategies are also a mechanism that can explain refereeing decisions. For instance, a preventive refereeing approach^33^ leads referees to applying the rules strictly early in the game, in order to build the expectations for the players that there will not be any leeway in their application. In another type of game management MacMahon and Mildenhall^34^ provide the example of a basketball referee calling an obviously incorrect foul on the visiting team which has a big lead in order to manage the volatile home crowd. In this case, the referee made the choice in favor of game management since the call was not deemed one that would materially change the outcome of the game. However, in a realistic scenario referees might change the process through which they are officiating dynamically. Raab et al.^35^ proposed a dynamic threshold model, where every referee has their own subjective threshold for game management. When the game reaches that threshold (e.g., via overly aggressive play from the two teams), the referee switches the underlying decision process mechanism from rule application to game management and vice versa. Focusing more on the reasons/processes behind the various referee bias, Bose et al.^22^ explored three potential mechanisms for referee bias towards elite soccer clubs in Germany. They considered career concerns (e.g., concerns that unfavorable calls for high prestige clubs will lead to exit from the league’s ranks), social pressure, as well as, effects from the performance of teams during their adolescence years. The study did not find evidence for any of these three mechanisms being drivers of the bias. In another interesting study, Morgulev et al.^31^ analyzed data from 500 instances of potential offensive fouls from the Israeli basketball league and studied the interaction between referee decision making and the decision for players to attempt to deceive the referees in order to get a favorable call. The authors found evidence that support the use of the representativeness heuristic^36^ by the referees to assess the situation. If a real offensive foul typically results in the defender falling, then the referee may have in mind this is the representative case, and he is more likely to not call an offensive foul when the defender does not fall.

While this work, unlike the studies above, does not deal with identifying the underlying mechanisms that lead to biased decision making, some of the findings could provide insights for other areas where similar implicit biases might appear. For example, referees of scientific work (e.g., research grants, research papers etc.) might also exhibit biases towards specific scientists and thus, policies/procedures should be put in place to avoid it (e.g., double blind reviews). As another example, the home court bias could potentially extend to areas like judicial trials (for which research has already shown other types of implicit biases). A judge who is familiar with a defense attorney (i.e., the attorney “plays” in home court, literally) may be more willing to listen carefully to the attorney’s arguments and motions. Here is were true randomization in judge/courthouse assignment can help, and this is exactly what US courts and courts abroad claim to do. However, there is evidence that the assignment is not always fully random. For example, Huther and Kleiner^37^ by analyzing bankruptcy fillings between 2010 and 2020 found that judge assignment is predicted by the lending decisions of hedge funds. Overall, while sports provide a controlled environment with a wealth of data that one can use to analyze and quantify these types of implicit biases, the lessons learned can be very useful in many different settings.

## Supplementary Information

Supplementary Information.

## Data Availability

The dataset used in this study, as well as, the scripts for their analysis are available at the following repository: https://github.com/kpelechrinis/NBA-ref-analysis.
