# Supplementary material for: Quantifying implicit biases in refereeing using NBA referees as a testbed
Source: Sci Rep. 2023 Mar 22;13:4664. doi: 10.1038/s41598-023-31799-y (PMC10031197; doi:10.1038/s41598-023-31799-y)
Supplement: Supplementary file 1 — Supplementary Information. [file 41598_2023_31799_MOESM1_ESM.pdf]

Supplementary Text 1: The use of technical foul data for assessing racial bias

In our analysis of potential racial bias we did not use the Last 2 Minutes (L2M) report, since they do not have the information necessary for the analysis of racial bias. In particular,

1. The L2M data do not provide information on which referee made a call, and therefore, it is not possible to create the racial pairs of referee-player needed. The analysis of the other types of biases examined (i.e., any potential home team bias, player bias and team bias), does not require the knowledge of which referee made or missed a call and, hence, L2M data are an appropriate data source to use for those.
2. Even if the referee making a call was mentioned in the L2M data this would account for a very small number of "mistakes". As shown in Figure 1, more than 95% of the calls are correct and hence, the data on incorrect calls would be very sparse. Most of the refereeing mistakes are violations that were missed and not called at all. And for these it is practically impossible to identify which referee was responsible for calling the infraction (e.g., the one closest to the play).

Given that this information was not available at the L2M data and to avoid – to the extent possible – the problems associated with not knowing whether a call for infraction was warranted or not (made or missed), we decided to rely on personal technical foul calls. Personal technical fouls are violations called from referees for "infractions of rules which do not involve physical activity". The most common reason for calling a technical foul is unsportsmanlike conduct, and they are highly subjective. For example, complaining to the referee during the game might result to a technical foul called by the referee but the same referee might exhibit different *threshold* for different players with regards to if/when to call an infraction. In fact, because they are subjective, the L2M reports do not review personal technical fouls assessed. Because of the subjective nature of this type of call we believe that it is a *good* substitute for the lack of L2M data that we can use.

Supplementary Table 1: Yearly recall for violations with at least 200 data points total

	violation	s2015	s2016	s2017	s2018	s2019	s2020	s2021	s2022
1	Turnover: Traveling	0.24	0.17	0.20	0.31	0.35	0.24	0.27	0.19
2	Foul: Personal	0.90	0.91	0.91	0.89	0.85	0.90	0.93	0.89
3	Foul: Shooting	0.85	0.84	0.83	0.77	0.76	0.74	0.81	0.75
4	Stoppage: Out-of-Bounds	1.00	1.00	1.00		0.75	0.60	1.00	0.98
5	Foul: Loose Ball	0.55	0.60	0.48	0.53	0.52	0.60	0.53	0.55
6	Instant Replay: Support Ruling	1.00	1.00	1.00	1.00	1.00	1.00		1.00
7	Foul: Offensive	0.35	0.28	0.41	0.38	0.33	0.45	0.54	0.52
8	Turnover: 24 Second Violation	0.84	0.98	1.00	0.98	1.00	0.95	0.97	0.98
9	Instant Replay: Overturn Ruling	1.00	1.00	1.00	1.00	1.00	1.00	1.00	1.00
10	Foul: Defense 3 Second	0.00	0.00	0.02	0.00	0.05	0.00	0.00	0.02
11	Foul: Personal Take		1.00	1.00	0.99	1.00	0.98	1.00	1.00
12	Turnover: Lost Ball Out of Bounds			1.00		0.98	0.97	0.97	0.99
13	Turnover: Out of Bounds - Bad Pass Turn					1.00	1.00	1.00	1.00

Supplementary Table 2: Yearly precision for violations with at least 200 data points total

	violation	s2015	s2016	s2017	s2018	s2019	s2020	s2021	s2022
1	Turnover: Traveling	0.93	0.90	0.81	0.89	0.91	0.78	0.88	0.87
2	Foul: Personal	0.98	0.96	0.99	0.98	0.98	0.97	0.96	0.97
3	Foul: Shooting	0.95	0.92	0.97	0.94	0.92	0.94	0.92	0.93
4	Stoppage: Out-of-Bounds	1.00	0.93	1.00	0.00	1.00	1.00	0.80	0.90
5	Foul: Loose Ball	0.94	0.95	0.95	0.97	0.99	0.94	0.93	0.97
6	Instant Replay: Support Ruling	1.00	1.00	1.00	1.00	1.00	1.00		1.00
7	Foul: Offensive	1.00	0.91	0.97	0.89	0.91	0.87	0.94	0.93
8	Turnover: 24 Second Violation	1.00	1.00	1.00	1.00	1.00	1.00	0.97	0.96
9	Instant Replay: Overturn Ruling	0.97	1.00	1.00	0.99	1.00	1.00	1.00	1.00
10	Foul: Defense 3 Second			1.00		1.00			1.00
11	Foul: Personal Take		1.00	1.00	1.00	1.00	1.00	0.99	1.00
12	Turnover: Lost Ball Out of Bounds			1.00		1.00	1.00	0.97	0.92
13	Turnover: Out of Bounds - Bad Pass Turn					1.00	1.00	1.00	0.98
